# Myelodysplastic Syndrome/Acute Myeloid Leukemia Following the Use of Poly-ADP Ribose Polymerase (PARP) Inhibitors: A Real-World Analysis of Postmarketing Surveillance Data

**DOI:** 10.3389/fphar.2022.912256

**Published:** 2022-06-15

**Authors:** Quanfeng Zhao, Pan Ma, Peishu Fu, Jiayu Wang, Kejing Wang, Lin Chen, Yang Yang

**Affiliations:** ^1^ Department of Pharmacy, The First Affiliated Hospital of Third Military Medical University (Army Medical University), Chongqing, China; ^2^ Department of Pharmacy, Women and Children’s Hospital of Chongqing Medical University, Chongqing, China; ^3^ Department of Pharmacy, Chongqing Health Center for Women and Children, Chongqing, China

**Keywords:** PARP inhibitors, myelodysplastic syndrome, acute myeloid leukemia, pharmacovigilance, real-world

## Abstract

**Background and purpose:** poly-ADP ribose polymerase (PARP) inhibitors show impressive efficacy in a range of tumors. However, concerns about rare and fatal adverse events, including myelodysplastic syndrome (MDS) and acute myelogenous leukemia (AML) have arisen. The aim of this study was to excavate and evaluate the risk of PARP inhibitors causing MDS and AML based on real-world data from two international pharmacovigilance databases.

**Methods:** We analyzed adverse event (AE) reports of four PARP inhibitors (olaparib, niraparib, rucaparib and talazoparib) associated with MDS and AML from the United States Food and Drug Administration (FDA) Adverse Event Reporting System (FAERS) and EudraVigilance (EV) databases between 1 October 2014, and 30 September 2021, including demographic characteristics, fatality and times to onset. Three different data mining algorithms were used to detect the signals of PARP inhibitors associated with MDS and AML.

**Results:** In total, 16,710 and 11,937 PARP inhibitor AE reports were found in the FAERS and EV databases, of which 332 and 349 were associated with MDS and AML, respectively. The median latencies of MDS and AML associated with PARP inhibitors were 211 [interquartile range (IQR) 93.5–491.25] days and 355 (IQR 72.00–483.50) days, respectively. The average fatality rates of MDS and AML caused by the four PARP inhibitors were 39.23 and 45.39%, respectively, in the FAERS database, while those in the EV database were 32.32 and 34.94%, respectively. Based on the criteria used for the three algorithms, a significant disproportionate association was found between PARP inhibitors as a drug class and MDS/AML. Notably, the risk of MDS was much higher than that of AML. Olaparib appeared to have a stronger association with MDS and AML than did other PARP inhibitors.

**Conclusion:** In the real world, PARP inhibitors increase the risk of MDS and AML, which can result in high mortality and tend to occur during long-term use. Our findings provide objective evidence for the postmarketing safety of PARP inhibitors.

## Introduction

In recent years, poly (ADP-ribose) polymerase (PARP) inhibitors, which rely on the mechanism of so-called synthetic sickness, have revolutionized the treatment of neoplasms, particularly in ovarian cancer (Lord and Ashworth, 2017). Four kinds of PARP inhibitors are approved by the Food and Drug Administration (FDA), including olaparib (Lynparza; AstraZeneca, initial FDA approval: December 2014), niraparib (Zejula, Tesaro, March 2017), rucaparib (Rubraca, Clovis Oncology, December 2016) and talazoparib (Talzenna, Pfizer, October 2018). A series of high-level evidence-based medical studies showed that PARP inhibitors have significant clinical benefits in patients with ovarian cancer (Coleman et al., 2017; Gonzalez-Martin et al., 2019; Ray-Coquard et al., 2019), breast cancer (Litton et al., 2018; Tutt et al., 2021), pancreatic cancer (Golan et al., 2019) and prostate cancer (Hussain et al., 2020).

However, with the increasing applications of PARP inhibitors, rare serious adverse reactions, especially myelodysplastic syndrome (MDS) and acute myeloid leukemia (AML), have become more prominent and are indicated by an FDA warning on the label. Although PARP inhibitors share the same mechanism of action, their specific toxicity profiles may vary considerably. Additionally, data from clinical trials showed low incidence rates of MDS and AML with PARP inhibitors, at between 0.5 and 1.4% (LaFargue et al., 2019). However, this measurement may be underestimated for assessing the association of PARP inhibitors with rare adverse events (AEs) because clinical trials have rigorous entry and exclusion criteria (such as excluding patients with higher burdens of comorbidities), relatively small sample sizes, and limited follow-up durations. Furthermore, the characteristics of MDS and AML caused by PARP inhibitors in the real world are poorly known. Therefore, the development of an understanding of their toxicity profiles by postmarketing pharmacovigilance is urgently needed.

The FDA Adverse Event Reporting System (FAERS) is the largest AE database in the world, containing more than 14 million reports. The FAERS database is considered the primary tool supporting the postmarketing safety surveillance of approved drugs and biologics. EudraVigilance (EV) is another public international spontaneously reported pharmacovigilance database for recording, managing and analyzing AEs in the European or non-European economic area and is maintained by the European Medicines Agency (EMA). Health care professionals, consumers, manufacturers and others can report AEs to the FAERS and EV databases. Analysis of the FAERS and EV databases provides a broader perspective for detecting AEs associated with newly approved drugs and rare AEs that occur in the real world (Pinheiro et al., 2016; Meng et al., 2019). The aim of this study was to characterize the association of PARP inhibitors (olaparib, rucaparib, niraparib, and talazoparib) with MDS/AML and to identify the signals of PARP inhibitor association with MDS/AML by utilizing real-world evidence.

## Methods

### Data Sources

Four PARP inhibitors namely (generic name) olaparib, rucaparib, niraparib and talazoparib were selected as study drugs (AE reports were only included if a target drug was listed as the primary suspect). Data were retrieved from the public release of FAERS and EV database between 1 October 2014 (considering the FDA approved the first PARP inhibitors, olaparib on 19 December 2014) and the 30 September 2021. OpenVigil FDA, a pharmacovigilance tool, is a web-based user interface to the FAERS database for extraction and analysis of adverse event safety reports which has been successful verified by FDA(Bohm et al., 2021). For the present study, we used the OpenVigil FDA to analysis the data from FAERS. AE reports of PARP inhibitors from EV database are publicly available through the EMA website (www.adrreports.eu). In accordance with the pharmacovigilance legislation, FDA and EMA operate procedures that ensure the quality and integrity of data collected in FAERS and EV database the AE files from FDA and EMA were updated every quarter.

### Definition of AEs

AEs are recoded using preferred terms (PTs) of the Medical Dictionary for Drug Regulatory Activities (MedDRA) terminology in FAERS and EV database. PT is a unique and clear expression in accordance with international standards for a single medical concept, and its specificity and description are strong. myelodysplastic syndrome (PT code: 10028533) and acute myeloid leukemia (PT code: 10000846) were selected as potential interest AEs for this study.

In this study, the inclusion criteria for AE report were that PARP inhibitor as “primary suspected drug”. Besides, we collected administrative and clinical characteristics of AE reports when data were available, including patient features (sex, age and country of origin), drug information (indication, concomitant drugs, therapy start dates and end dates), and final patient outcomes. We removed duplicated and aberrant reports (such as the date of adverse event occurrence is earlier than the start time of medication).

### Statistical Analysis

Disproportionality analyses were commonly used to identify potential safety signals for AEs in the FAERS, including the established pharmacovigilance algorithms reporting odds ratio (ROR), proportional reporting ratio (PRR) and Bayesian confidence propagation neural network (BCPNN). Each method has its own characteristics, ROR or PRR algorithm has high sensitivity, but it is easy to produce false positive signals when the number of AE reports is not enough (Rothman et al., 2004). Bayesian algorithm has good stability, but the signal detection time is lagged (Bate, 2007). In order to reduce the bias caused by using a single algorithm as much as possible, three different data mining algorithms (ROR, PRR and BCPNN) were used for signal detection in this study. When all the three algorithms are positive, the signal is judged as suspicious AE signal. the equations and criteria for the three algorithms are shown in Supplementary Table S2, which is based on the fourfold table of disproportionality measurement (Supplementary Table S1).

Only FAERS can realize signal detection by using open database gratuitous, Therefore, we detected the signal value of PARP inhibitors associated with MDS and AML only in FAERS. Moreover, we summarized the time to onset for AEs of interest only in EV database due to data limitation and the analysis was conducted by GraphPad Prism (version 8.3.0).

## Results

### Descriptive Analysis

During the study period, a total of 16,710 AE reports corresponding to PARP inhibitors were extracted from the FAERS database, including 5670 for olaparib, 8211 for niraparib, 2475 for rucaparib and 354 for talazoparib. Among these cases, the total numbers of MDS and AML AE cases were 187 and 145, respectively. In addition, the EV database presented a total of 11,937 events corresponding to PARP inhibitors, including 5493 for olaparib, 4854 for niraparib, 1428 for rucaparib and 198 for talazoparib. The total numbers of potential AEs of interest for PARP inhibitors were 222 and 127. The most frequently reported drug in the two databases was olaparib. Table 1 shows the characteristics for MDS reported with PARP inhibitors in the two databases, and Table 2 shows the same for AML. In these PARP associated with MDS/AML cases, we noted cases exposed to PARP inhibitors were similar between the European economic area and non-European economic area. More than 80% PARP inhibitors were used for ovarian cancer treatment(Table 3), and the patient gender of MDS and AML caused by the four PARP inhibitors were 97.6 and 91.73%, respectively, in the FAERS database, while those in EV database were 98.64 and 92.56%.

**TABLE 1:** The characteristics of MDS reported by PARP inhibitors in FAERS and EV database.

	Olaparib		Niraparib		Rucaparib		Talazoparib		Total	
	FAERS	EV	FAERS	EV	FAERS	EV	FAERS	EV	FAERS	EV
**Total cases**	147	179	29	32	7	10	4	1	187	222
**Age**										
<18 y	0 (0)	0 (0)	0 (0)	0 (0)	0 (0)	0 (0)	0 (0)	0 (0)	0 (0)	0 (0)
18−64 y	50 (60.98)	75 (60.98)	8 (53.33)	8 (57.14)	1 (25.00)	4 (66.67)	1 (25.00)	0 (0)	60 (57.14)	87 (60.42)
65−85 y	31 (37.80)	47 (38.21)	7 (46.67)	6 (42.86)	3 (75.00)	2 (33.33)	3 (75.00)	1 (100.00)	44 (41.90)	56 (38.89)
>85 y	1 (1.22)	1 (0.81)	0 (0)	0 (0)	0 (0)	0 (0)	0 (0)	0 (0)	1 (0.95)	1 (0.69)
data available	82	123	15	14	4	6	4	1	105	144
**Gender**										
female	136 (99.27)	177 (99.44)	21 (95.45)	31 (96.88)	3 (75.00)	9 (90.00)	3 (75.00)	1 (100.00)	163 (97.60)	218 (98.64)
male	1 (0.73)	1 (0.56)	1 (4.55)	1 (3.13)	1 (25.00)	1 (10.00)	1 (25.00)	0 (0)	4 (2.40)	3 (1.36)
data available	137	178	22	32	4	10	4	1	167	221
**Reporting region**										
European Economic Area	68 (46.26)	89 (49.72)	11 (37.93)	18 (56.25)	4 (57.14)	3 (30.00)	3 (75.00)	1(100.00)	86(45.99)	111(50%)
Non-European Economic Area	79(53.74)	90(50.28)	18(62.07)	14(43.75)	3(42.86)	7(70.00)	1(25.00)	0(0)	101(54.01)	111(50%)
data available	147	179	29	32	7	10	4	1	187	222
**Indication**										
ovarian cancer	105(85.37)	134(93.06)	22(88.00)	18(90.00)	2(50.00)	3(75.00)	0(0)	0(0)	129(82.69)	155(91.72)
breast cancer	8(6.50)	5(3.47)	1(4.00)	1(5.00)	0(0)	0(0)	2(50.00)	1(100.00)	11(7.05)	7(4.14)
pancreatic carcinoma	2(1.63)	0(0)	0(0)	0(0)	0(0)	0(0)	0(0)	0(0)	2(1.28)	0(0)
prostate cancer	1(0.81)	0(0)	0(0)	0(0)	0(0)	0(0)	2(50.00)	0(0)	3(1.92)	0(0)
other malignant neoplasm	7(5.69)	5(3.47)	2(8.00)	1(5.00)	2(50.00)	1(25.00)	0(0)	0(0)	11(7.05)	7(4.14)
data available	123	144	25	20	4	4	4	1	156	169

**TABLE 2:** The characteristics of AML reported by PARP inhibitors in FAERS and EV database.

	Olaparib		Niraparib		Rucaparib		Talazoparib		Total	
	FAERS	EV	FAERS	EV	FAERS	EV	FAERS	EV	FAERS	EV
**Total cases**	110	103	30	19	4	5	1	0	145	127
**Age**										
<18 y	1(1.33)	1(1.47)	0(0)	0(0)	0(0)	0(0)	0(0)	0(0)	1(0.99)	1(1.20)
18–64 y	42(56.00)	29(42.65)	15(65.22)	9(69.23)	2(100.00)	0(0)	1(100.00)	0(0)	60(59.41)	38(45.78)
65–85 y	30(40.00)	36(52.94)	7(30.43)	4(30.77)	0(0)	2	0(0)	0(0)	37(36.63)	42(50.60)
>85 y	2(2.67)	2(2.94)	1(4.35)	0(0)	0(0)	0(0)	0(0)	0(0)	3(2.97)	2(2.41)
data available	75	68	23	13	2	2	1	0	101	83
**Gender**										
female	94(91.26)	95(93.14)	23(92.00)	12(85.71)	4(100.00)	5(100.00)	1(100.00)	0(0)	122(91.73)	112(92.56)
male	9(8.74)	7(6.86)	2(8.00)	2(14.29)	0(0)	0(0)	0(0)	0(0)	11(8.27)	9(7.44)
data available	103	102	25	14	4	5	1	0	133	121
**Reporting region**										
European Economic Area	53(48.18)	56(54.37)	17(56.67)	9(47.37)	1(25.00)	0(0)	1(100.00)	0(0)	72(49.66)	65(51.18)
Non-European Economic Area	57(51.82)	47(45.63)	13(43.33)	10(52.63)	3(75.00)	5(100.00)	0(0)	0(0)	73(50.34)	62(48.82)
data available	110	103	30	19	4	5	4	0	145	127
**Indication**										
ovarian cancer	77(81.05)	77(89.53)	18(90.00)	11(100.00)	2(100.00)	1(100.00)	0(0)	0(0)	97(82.20)	89(90.82)
breast cancer	3(3.16)	3(3.49)	0(0)	0(0)	0(0)	0(0)	1(100.00)	0(0)	4(3.39)	3(3.06)
prostate cancer	6(6.32)	6(6.98)	0(0)	0(0)	0(0)	0(0)	0(0)	0(0)	6(5.08)	6(6.12)
other malignant neoplasm	9(9.47)	0(0)	2(10.00)	0(0)	0(0)	0(0)	0(0)	0(0)	11(9.32)	0(0)
data available	95	86	20	11	2	1	1	0	118	98

**TABLE 3:** Top 3 concomitant medications for PARP inhibitors associated with MDS and AML from FAERS and EV databases.

	Olaparib(N^a^)		Niraparib(N)		Rucaparib(N)		Talazoparib(N)	
	FAERS	EV	FAERS	EV	FAERS	EV	FAERS	EV
MDS	Carboplatin (70)	Carboplatin (31)	Carboplatin (14)	Rivaroxaban (5)	Carboplatin (2)	Ondansetron (2)	—	—
	Paclitaxel (47)	Paclitaxel (26)	Doxorubicin (13)	Carboplatin (2)	paclitaxel (1)	Colecalciferol (2)	—	—
	Doxorubicin (26)	Bevacizumab (10)	Cisplatin (7)	Doxorubicin (2)	Gabapentin (1)	Bevacizumab (1)	—	—
AML	Carboplatin (28)	Carboplatin (32)	Acetaminophen (6)	Doxorubicin (4)	Acetaminophen (6)	Melatonin (1)	Enzalutamide (1)	—
	Paclitaxel (13)	Paclitaxel (23	Budesonide and formoterol fumarate (6)	Carboplatin (2)	Azacytidine (1)	—	—	—
	Bevacizumab (9)	Bevacizumab (15)	Calcium and vitamin D (4)	Cisplatin (1)	Binpcrit (1)	—	—	—

a If the same number of cases are encountered, they are showed in alphabetical order.

### Outcome of MDS and AML Reported By PARP Inhibitors in Two Databases

To determine the fatal risk, we measured the mortality rates of MDS and AML reported along with the four targeted PARP inhibitors in the two databases, and the generated results are shown in Table 4. We found that the mortality rate in the FAERS database was higher than that in the EV database; the universal mortality rates of MDS and AML caused by the four PARP inhibitors in the FAERS database were 39.23 and 45.39%, while those in the EV database were 32.32 and 34.94%, respectively.

**TABLE 4:** The serious outcome of MDS and AML reported by PARP inhibitors in FAERS and EV database^a^.

	Olaparib		Niraparib		Rucaparib		Talazoparib		Total	
	MDS	AML	MDS	AML	MDS	AML	MDS	AML	MDS	AML
**FAERS**	147	110	29	30	7	4	4	1	187	145
death	36(41.38)	50(48.54)	10(33.33)	16(39.02)	3(42.86)	2(40.00)	2(33.33)	1(33.33)	51(39.23)	69(45.39)
life-threatening	26(29.89)	27(26.21)	11(36.67)	19(46.34)	1(14.86)	1(20.00)	1(16.67)	1(33.33)	39(30.00)	48(31.58)
hospital prolonged	23(26.44)	25(24.27)	7(23.33)	5(12.20)	3(42.86)	2(40.00)	3(50.00)	1(33.33)	36(27.69)	33(21.71)
disablity	2(2.30)	1(0.97)	2(6.67)	1(2.44)	0(0.00)	0(0.00)	0(0.00)	0(0.00)	4(3.07)	2(1.32)
data available	87	103	30	41	7	5	6	3	130	152
**EV**	179	103	32	19	10	5	4	1	222	127
death	27(36.49)	24(34.78)	4(19.05)	5(35.71)	1(33.33)	0(0.00)	0(0.00)	0(0.00)	32(32.32)	29(34.94)
life-threatening	25(33.78)	21(30.43)	7(33.33)	4(28.57)	0(0.00)	0(0.00)	0(0.00)	0(0.00)	32(32.32)	25(30.12)
hospital prolonged	18(24.32)	22(31.88)	9(42.86)	5(35.71)	2(66.67)	0(0.00)	1(100.00)	0(0.00)	30(30.30)	27(32.53)
disablity	4(5.41)	2(2.90)	1(4.76)	0(0.00)	0(0.00)	0(0.00)	0(0.00)	0(0.00)	5(5.51)	2(2.41)
data available	74	69	21	14	3	0	1	0	99	83

a Multiple outcomes can be reported for the same report.

### Time to Onset of MDS and AML Reported By PARP Inhibitors in EV Databases

**FIGURE 1:**
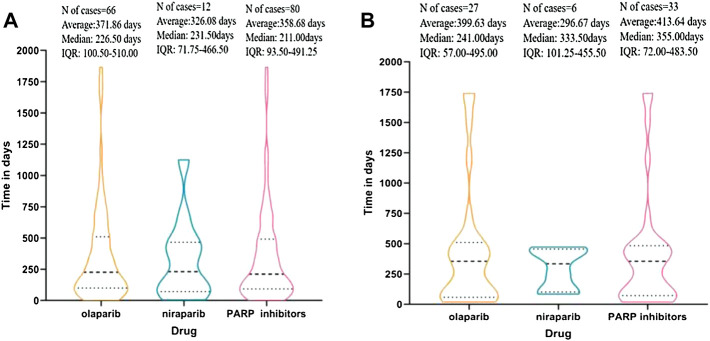
**(A)** Time to event onset of myelodysplastic syndrome (MDS) following olaparib and niraparib. **(B)** Time to event onset of acute myeloid leukemia (AML) following olaparib and niraparib. Data on rucaparib and talazoparib are not available since the missing data (number of cases < 4).

Overall, the median times to all PARP inhibitor-related MDS and AML events were 211 [interquartile range (IQR) 93.50–491.25] days and 355 (IQR 72.00–483.50) days, respectively. We display the time to onset of MDS/AML events for each PARP inhibitor in Figures 1A,B. The times to occurrence of MDS/AML ranged from the first days or weeks to 1 year or more after the start of therapy, with most times concentrated within 1 year. Because of the scantness of data, the times to MDS and AML event were not computed for rucaparib and talazoparib.

### Disproportionality Analysis of MDS/AML Associated With PARP Inhibitors

**FIGURE 2:**
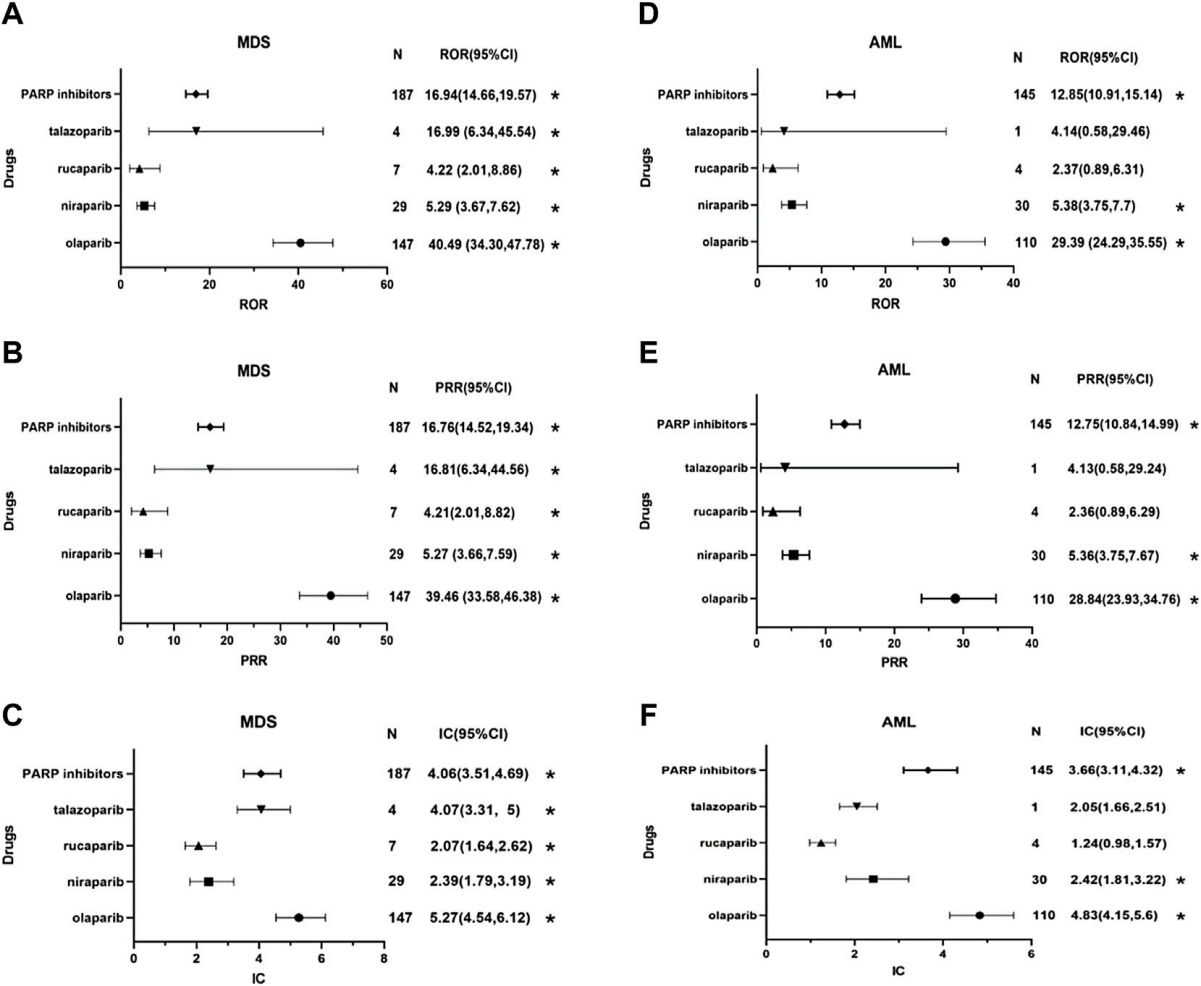
Forest plots of disproportionality analysis of MDS/AML associated with PARP inhibitors. * Statistically significant disproportionality (the standard of signal detection in the Supplementary Table S2). Abbreviation: MDS, myelodysplastic syndrome; AML, acute myeloid leukemia; N, the number of reports of PARP-associated myelodysplastic syndrome or acute myeloid leukemia; CI, confidence interval; ROR, reporting odds ratio; PRR, proportional reporting ratio; IC, information component; PARP inhibitors, poly (ADP-ribose) polymerase inhibitors. **(A)** the reporting odds ratio (ROR) distribution of MDS reported by PARP inhibitors. **(B)** the proportional reporting ratio (PRR) distribution of MDS reported by PARP inhibitors. **(C)** the information component (IC) distribution of MDS reported by PARP inhibitors. **(D)** the reporting odds ratio (ROR) distribution of AML reported by PARP inhibitors. **(E)** the proportional reporting ratio (PRR) distribution of AML reported by PARP inhibitors. **(F)** the information component (IC) distribution of AML reported by PARP inhibitors.

Based on the criteria used for the three algorithms in the FAERS database, a significant disproportionate association was found between PARP inhibitors as a drug class and MDS and AML [**MDS**: 16.94 (14.66–19.57) for ROR, 16.76 (14.52–19.34) for PRR and 4.06 (3.51–4.69) for IC; **AML**: 12.85 (10.91–15.14) for ROR, 12.75 (10.84–14.99) for PRR and 3.66 (3.11–4.32) for IC]. For each PARP inhibitor, the association results are showed in Figure 2. The signal scores suggest that all four PARP inhibitors are associated with MDS. The relationship of olaparib with MDS was noteworthy due to having the highest ROR, PRR and BCPNN values [40.49 (34.3–47.78) for ROR, 39.46 (33.58–46.38) for PRR and 5.27 (4.54–6.12) for IC], whereas the signal values of rucaparib-related MDS were the weakest [4.22 (2.01–8.86) for ROR, 4.21 (2.01–8.82) for PRR and 2.07 (1.64–2.62) for IC]. Concerning AML, the signal scores suggest that only olaparib and niraparib are associated with AML, whereas no significant signals were detected for rucaparib (the 95% CI of ROR and PRR value is lower than 1) and talazoparib (the 95% CI of ROR and PRR value is lower than 1 and the cases of interest reported is lower than 3; the standard of signal detection are shown in Supplementary Tables S1, S2). Olaparib also had the highest signal values for AML [29.39 (24.29–35.55) for ROR, 28.84 (23.93–34.76) for PRR and 4.83 (4.15–5.6) for IC].

## Discussion

PARP inhibitors that are in the same category of drugs show some of the same toxic characteristics, but different drugs have different adverse reactions, and they vary in frequency and severity (Staropoli et al., 2018). Previous studies have shown that common toxicities among PARP inhibitors include hematological toxicity, gastrointestinal adverse reactions, nephrotoxicity, and fatigue, which usually occur during the first 3 months of treatment (LaFargue et al., 2019). However, a few randomized controlled trials (RCTs) have reported MDS and AML in patients using PARP inhibitors and indicated possible delayed toxicity of PARP inhibitor therapy (Mirza et al., 2016; Coleman et al., 2017).

To the best of our knowledge, this is the first pharmacovigilant analysis of MDS and AML adverse events associated with PARP inhibitors utilizing the FAERS and EV databases. Myelodysplastic syndrome and acute leukemia, these rare, serious and delayed adverse reactions, are the most concerning adverse reactions associated with PARP inhibitors therapy. Current literature reveals inconsistent conclusions about the associations of PARP inhibitors with MDS and AML. A meta-analysis of 5739 patients from 14 RCTs showed no significant association between PARP inhibitors and the incidence rates of MDS and AML (Nitecki et al., 2021). In some RCTs or meta-analyses, rare or delayed adverse events were not fully captured during short-term follow-up and may be affected by the size of the dataset. On the other hand, the final analysis of SOLO-2, which is the largest related long-term follow-up study that has been conducted, suggested that olaparib may cause MDS and AML (Poveda et al., 2021). A meta-analysis of 9099 patients from 28 RCTs and a retrospective study of the World Health Organization (WHO) pharmacovigilance database demonstrated that the combination of PARP inhibitors significantly increased the risk of MDS and AML (Peto OR 2.63 [95% CI 1·13–6·14], *p* = 0·026) (Morice et al., 2021). In another study, an increased risk of MDS/AML was observed in real-world patients who received PARP inhibitors (Ma et al., 2021; Matsuo et al., 2021). Our findings are consistent with the results of these epidemiological studies and meta-analyses. In addition, we have enumerated the following notable and interesting findings:

First, the risk of MDS related to PARP inhibitors was much higher than that of AML. MDS shares the clinical and pathological features of AML but shows a lower percentage of primitive cells in the peripheral blood and bone marrow (<20%) (Arber et al., 2016). Patients with MDS may develop symptomatic anemia, infection, and bleeding and may transition to having AML, and the incidence rates of MDS and AML are also different (Siegel et al., 2012; McQuilten et al., 2014). Our findings indicate that PARP inhibitors are associated with a higher risk of MDS than AML in a “real world” setting. According to the results of the meta-analysis involving several RCTs, there were no significant differences between the risks of MDS and AML associated with PARP inhibitors, which may account for differences between the real world and RCTs with rigorous study entry criteria (Morice et al., 2021). Indeed, a previous study showed that the power of RCTs to detect AEs was weak, especially for rarer AEs (Salem et al., 2021). Additional analysis is necessary based on the observed differences between RCTs and the “real world”.

Second, MDS and AML signals were detected for PARP inhibitors as a drug class included in this study, suggesting that MDS and AML may be common AEs to PARP inhibitors. However, rucaparib and talazoparib-related AML showed no positive signals, which may be related to the short marketing time of these PARP inhibitors, and further studies on the risk of AEs associated with rucaparib and talazoparib are needed. Poly (ADP-ribose) polymerases are a family of enzymes that use the oxidized form of nicotinamide adenine dinucleotide to transfer ADP-ribose to other proteins (poly ADP-ribosylation). They are involved in deoxyribonucleic acid (DNA) damage response, regulation of apoptosis, and maintenance of genomic stability (Anderson et al., 2016). The anti-cancer mechanism of PARP inhibitors mainly includes two factors. PARP inhibitors can inhibit the activity of PARP enzymes and prevent DNA single-strand repair. PARP inhibitors can also stabilize the structure of DNA-PARP complexes and hinder their separation. This process, also known as trapping, results in long-term existence of DNA-PARP complexes that inhibit subsequent DNA repair processes (Murai et al., 2014). The exact mechanisms by which PARP inhibitor induces MDS and AML are unknown and may be multifaceted. Myeloid neoplasms, including MDS and AML, are heterogeneous diseases with multiple potential molecular abnormalities, characterized by high chromosomal instability, which is considered to be caused by the wrong DNA damage repair mechanism (Esposito and So, 2014). PARP family proteins play an important role in maintaining hematopoietic function, and the regulation of the expression of PARP family proteins differs between acute myelogenous leukemia cells and healthy cells (Gil-Kulik et al., 2020). PARP inhibitors can lead to acquired mutations with clonal hematopoiesis in the circulatory system through DNA-damaging reactions, thereby increasing the risk of MDS and AML. Additionally, PARP inhibitors may also cause off-target epigenetic changes that can result in MDS and AML through potential clonal hematopoietic transformations (Bolton et al., 2020). Besides, there were interactions between breast cancer susceptibility protein 1 or 2 (BRCA1 or BRCA 2) mutations and Fanconi Anemia proteins in the homologous recombination pathway. Some data suggest that BRCA deficiency may increase the risk of MDS/AML (Friedenson, 2007). These molecular studies might help explain some of the myeloid symptoms reported by patients using PARP inhibitors. Furthermore, concomitant medications should also be considered because the use of alkylating agents, topoisomerase inhibitors, platinum drugs, and bevacizumab has been reported to significantly increase the risk of MDS/AML (Shenolikar et al., 2018; Morton et al., 2019). According to information in the FAERS and EV databases, PARP inhibitors were mostly used for ovarian cancer treatment, and the common concomitant medications for PARP inhibitors were carboplatin and paclitaxel. Platinum-and-paclitaxel-based chemotherapy is the standard first-line chemotherapeutic regimen for ovarian cancer. Therefore, we cannot rule out the possibility that the combination of PARP inhibitors and chemotherapy drugs might increase the risk of MDS and AML. Overall, the mechanism of PARP inhibitors causing MDS/AML still needs to be further studied by relevant studies.

In addition, the results of the three signal mining methods showed that olaparib had the highest signal values for MDS and AML compared with those of other PARP inhibitors, indicating that olaparib had stronger associations with MDS and AML. it is difficult to explain the discrepancies between various PARP inhibitors, cases of olaparib-induced MDS have been noted in clinical practice (Moore et al., 2018). Some insights on the pharmacodynamic characteristics of PARP inhibitors have shown that olaparib among PARP inhibitors has a submifromolar potency on the PARP protein family (Thorsell et al., 2017), as increased PARP trapping has been proved to be associated with high myelosuppression (Hopkins et al., 2019). It remains to be further confirmed whether these characteristics of olaparib are likely to be highly correlated with MDS and AML, and the head-to-head comparison clinical studies are needed in the future.

Third, we observed that the prognoses of MDS and AML were not optimistic, leading to as high as 30% mortality and life-threatening rates, especially the universal mortality rates was 39.23 and 45.39% in the FAERS database. This result is similar to the finding reported in a recent study based on the WHO pharmacovigilance database (45.2%) (Morice et al., 2021). Even more worrisome, a SEER study reported that MDS/AML-related mortality can be as high as 78% (Morton et al., 2019). We should increase clinical vigilance regarding PARP inhibitor associations with MDS and AML. If the patient has persistent cytopenias, further investigations are recommended, including bone marrow analysis and blood samples for cytogenetics, and consideration of discontinuation of PARP inhibitors. If MDS or AML is confirmed, PARP inhibitors must be discontinued. American Society of Clinical Oncology guidelines also suggest that evaluations of treatment related MDS and AML should be initiated in patients with persistent cytopenia despite drug withdrawal (Tew et al., 2020).

Fourth, the timing of AE occurrence varies for each PARP inhibitor, and our study indicated that the risk of MDS/AML usually emerged after long-term treatment, which is in line with the results of previous studies (Mirza et al., 2016; Pujade-Lauraine et al., 2017). Care should be taken when prescribing these drugs for long-term use. Specifically, the first 1 year can be considered the “critical pharmacovigilance window” for olaparib and niraparib.

In this study, the detection signals of PARP inhibitor-related MDS and AML in two international pharmacovigilance databases were analyzed by the ROR, PRR and BCPNN methods, which can reflect the safety of drugs in the real world to a certain extent. However, we acknowledge several inherent limitations in our study. First, the incidence of adverse events could not be calculated due to the lack of overall drug use data. Second, although ROR, PRR and BCPNN are quantitative signal detection methods, they are only simple indicators of potential safety problems and can only indicate whether there is statistical correlation between drugs and AEs. Third, the spontaneous AE reports in the databases are arbitrary and biased and can include characteristics such as underreporting and missing information, which could also have affected the results.

Despite these limitations, the findings of this study indicate potential safety problems regarding the development of MDS and AML when using PARP inhibitors. Such adverse reactions are rare but lethal, and these results can provide a reference for clinical workers in the use of PARP inhibitors.

## Conclusion

In this study, the results indicated that the use of PARP inhibitors may lead to MDS and AML toxicity, and the potential associations of olaparib were stronger. In addition, MDS and AML often occur in patients with long-term medication use, and their mortality rates are high. It was suggested that clinicians should pay more attention to the risk of MDS and AML when using PARP inhibitors. Our findings provide objective evidence for the postmarketing safety of PARP inhibitors.

## Data Availability

The datasets presented in this study can be found in online repositories. The names of the repository/repositories and accession number(s) can be found below: http://openvigil.sourceforge.net/; https://www.adrreports.eu/.

## Abbreviations

AE, adverse event; AML, Acute myeloid leukemia; BCPNN, Bayesian confidence propagation neural network; BRCA, breast cancer susceptibility protein; DNA, deoxyribonucleic acid; EV, EudraVigilance; EMA, European Medicines Agency; FDA, Food and Drug Administration; FAERS, FDA Adverse Event Reporting System; IQR, interquartile range; MedDRA, Medical Dictionary for Drug Regulatory Activities; MDS, Myelodysplastic syndrome; PARP, poly-ADP ribose polymerase; PRR, proportional reporting ratio; PT, preferred terms; RCT, randomized controlled trials; ROR, reporting odds ratio; WHO, World Health Organization.
