# Corrigendum: Myelodysplastic syndrome/acute myeloid leukemia following the use of poly-ADP ribose polymerase inhibitors: A real-world analysis of postmarketing surveillance data

**DOI:** 10.3389/fphar.2022.990048

**Published:** 2022-08-11

**Authors:** Quanfeng Zhao, Pan Ma, Peishu Fu, Jiayu Wang, Kejing Wang, Lin Chen, Yang Yang

**Affiliations:** ^1^ Department of Pharmacy, The First Affiliated Hospital of Third Military Medical University (Army Medical University), Chongqing, China; ^2^ Department of Pharmacy, Women and Children’s Hospital of Chongqing Medical University, Chongqing, China; ^3^ Department of Pharmacy, Chongqing Health Center for Women and Children, Chongqing, China

**Keywords:** PARP inhibitors, myelodysplastic syndrome, acute myeloid leukemia, pharmacovigilance, real-world

Text Correction

In the original article, there was an error. Several result values were written incorrectly in the article’s abstract. The corrected **abstract** appears below:

In total, 16,710 and 11,937 PARP inhibitor AE reports were found in the FAERS and EV databases, of which 332 and 349 were associated with MDS and AML, respectively. The median latencies of MDS and AML associated with PARP inhibitors were 211 [interquartile range (IQR) 93.5–491.25] days and 355 (IQR 72.00–483.50) days, respectively. The average fatality rates of MDS and AML caused by the four PARP inhibitors were **39.23 and 45.39%**, respectively, in the FAERS database, while those in the EV database were **32.32 and 34.94%**, respectively. Based on the criteria used for the three algorithms, a significant disproportionate association was found between PARP inhibitors as a drug class and MDS/AML. Notably, the risk of MDS was much higher than that of AML. Olaparib appeared to have a stronger association with MDS and AML than did other PARP inhibitors.

The authors apologize for this error and state that this does not change the scientific conclusions of the article in any way. The original article has been updated.

