# Supplementary material for: Myelodysplastic Syndrome/Acute Myeloid Leukemia Following the Use of Poly-ADP Ribose Polymerase (PARP) Inhibitors: A Real-World Analysis of Postmarketing Surveillance Data
Source: Front Pharmacol. 2022 Jun 15;13:912256. doi: 10.3389/fphar.2022.912256 (PMC9240214; doi:10.3389/fphar.2022.912256)
Supplement: Supplementary file 1 [file Table1.docx]

**Supplementary Data**

**Table S1. Fourfold table of disproportionality measurement**

|  | Drug of interest | All Other drugs of interest | Total |
| --- | --- | --- | --- |
| Adverse event of interest | A | B | A+B |
| All other events of interest | C | D | C+D |
| Total | A+C | B+D | A+B+C+D |

**Table S2. Algorithms of disproportionality analysis and standard of signal detection**

| Algorithms | Calculation formula | Criteria |
| --- | --- | --- |
| ROR | ROR= $\frac{A/C}{B/D}$  $95\%CI=e^{\ln(ROR)\pm1.96\sqrt{(\frac{1}{A}+\frac{1}{B}+\frac{1}{C}+\frac{1}{D})}}$ | A≥3; 95%CI＞1 |
| PRR | PRR= $\frac{A/(A+B)}{C/(C+D)}$  $95\%CI=e^{\ln(PRR)\pm1.96\sqrt{(\frac{1}{A} \frac{1}{A+B}+\frac{1}{C} \frac{1}{C+D})}}$ | A≥3; 95%CI＞1 |
| BCPNN | IC=$\log_{2} A(A+B+C+D)(A+C)(A+B)$ $95\%CI=e^{\ln(IC)\pm1.96\sqrt{(\frac{1}{A}+\frac{1}{B}+\frac{1}{C}+\frac{1}{D})}}$ | IC025 > 0 |

Abbreviations: ROR: reporting odds ratio; PRR: proportional reporting ratio; BCPNN: Bayesian confidence propagation neural networks; IC: information component; 95%CI: 95% confidence interval; IC025: the lower limit of the 95% two-sided confidence interval of the information component.
